# Exploring the feasibility of Recovery Management Checkups for Primary Care in a Federally Qualified Health Center

**DOI:** 10.3389/fpubh.2024.1443409

**Published:** 2024-11-11

**Authors:** Dennis P. Watson, Ryan Singh, Lisa Taylor, Michael L. Dennis, Christine E. Grella, Carol Johnstone, Katherine Browne, Lisa Saldana

**Affiliations:** ^1^Lighthouse Institute, Chestnut Health Systems, Chicago, IL, United States; ^2^Johnstone Consulting, LLC, Chicago, IL, United States; ^3^Independent Contractor, Bloomington, IL, United States

**Keywords:** recovery, treatment linkage, feasibility, primary care, substance use disorder

## Abstract

**Introduction:**

Primary care settings present an opportunity for alcohol and substance use disorder (A/SUD) screening and treatment referral. However, there are recognized deficiencies in widely used treatment referral approaches, including acute care connections, vs. those that can support longer-term recovery. Recovery Management Checkups for Primary Care (RMC-PC) is an intervention with an evidence base for improving treatment referral and subsequent recovery for primary care patients; however, the intervention has never been fully implemented outside of a research context. We conducted a feasibility study to inform a future hybrid study of RMC-PC that will test the implementation and effectiveness of the intervention in primary care practice.

**Method:**

We used a convergent mixed method design. The study’s setting was a Federally Qualified Health Center (FQHC) located in a large midwestern city. RMC-PC linkage services were administered by one of two treatment linkage managers: an FQHC linkage manager (F-LM) and a research staff linkage manager (R-LM). Quantitative data included (a) rates of positive A/SUD screening among a group of FQHC patients and (b) linkage manager service data (e.g., rate of successful meeting completion and days to completing of key events). Qualitative data included (c) an assessment of linkage manager’s motivational interviewing performance and (d) a focus group with FQHC staff focused on their perspectives on RMC-PC implementation determinants. Quantitative data were summarized using descriptive statistics, and linkage manager performance was compared. Qualitative data were analyzed using a hybrid deductive-inductive process.

**Results:**

Fifty percent of patients screened met moderate-high A/SUD risk. Eleven of 16 recruited patients completed at least one linkage manager meeting, with 63% completing both meetings. The F-LM delivered RMC-PC services alongside other duties successfully; however, three primary barriers to FQHC implementation were identified (difficulties applying motivational interviewing, incompatibilities of screening with FQHC technology and workflow, and lack of billing mechanism to support services).

**Conclusion:**

RMC-PC is feasible for FQHC staff to deliver, though issues identified must be considered to ensure successful and sustainable implementation. Knowledge gained will inform a packaged implementation strategy that will be used in a future hybrid trial.

## 1 Introduction

Among the approximately 54.6 million people identified as needing treatment for an alcohol or substance use disorder (A/SUD) in the United States in 2022, less than one-quarter actually received it (1). Primary care settings present an opportunity for A/SUD recognition and referral to services and treatment necessary to initiate and support long-term recovery. Indeed, primary care settings are recommended to use Screening, Brief Intervention, and Referral to Treatment (SBIRT) to accomplish this task (2). SBIRT is a three-step process— (1) A/SUD screening, (2) short motivational intervention, and (3) referring patients with higher level need to treatment. Although there is evidence of SBIRT’s effectiveness for identifying A/SUD and reducing alcohol consumption in at-risk drinkers (3–6), its ability to improve treatment referral and longer-term treatment and recovery outcomes for those with more serious levels of misuse is lacking (5, 7). Recovery Management Checkups (RMC) is an intervention for facilitating A/SUD treatment linkage, engagement, and care continuity with an evidence base consisting of four clinical trials conducted over two decades (8–13). The most recent randomized trial of RMC, tailored for primary care (RMC-PC) and delivered to patients recruited from four Federally Qualified Health Centers (FQHC), demonstrated the effectiveness of SBIRT+RMC-PC compared to SBIRT alone (12–14). The observed benefits of RMC-PC over 12 months included significantly greater treatment linkage odds, more days of treatment and abstinence, and fewer days of alcohol use, cannabis use, and overall substance use (13). RMC-PC holds considerable promise for addressing SBIRT’s recognized referral limitation and supporting longer-term treatment engagement and recovery. Also, while implemented as an adjunct to SBIRT in the previous clinical trial, prior trials (8–11) demonstrate RMC-PC does not require SBIRT and can operate using any functional screening mechanism. However, the intervention has never been fully implemented, with or without SBIRT, outside of a research context, making the extent to which primary care staff can deliver RMC-PC successfully under real-world conditions uncertain. This article describes the results of a study we undertook immediately after completing the RMC-PC trial to explore the feasibility of RMC-PC delivery by FQHC primary care staff.

RMC-PC is a treatment linkage and recovery support intervention provided to patients in need of referral to brief or more intensive treatment options (patients identified with mild substance misuse not requiring treatment are ineligible for the intervention in its current form). Guided by a chronic disease model, it relies on assertive protocols to facilitate patient treatment linkage and ongoing monitoring through checkups that provide early detection and re-intervention before the relapse becomes severe or sustained (10, 15, 16). The entire RMC-PC intervention lasts 1 year. Once screening identifies a person with a potential A/SUD treatment linkage need, an assessment is completed to understand their substance use and how it impacts their lives, their motivation to stop using substances, importance of recovery, and potential barriers to treatment. This information is then translated into a linkage worksheet that a linkage manager uses to guide a motivational interviewing (MI) discussion focused on helping patients resolve any identified ambivalence regarding the problematic aspects of their substance use and facilitating a commitment to change through accessing treatment or alternative strategies. The linkage manager also identifies and addresses any treatment barriers, develops a treatment linkage plan, and uses frequent check-ins to assist the patient in completing the linkage plan and supports treatment engagement and continuity of care/aftercare. A more formal checkup that includes a re-assessment and linkage worksheet-guided MI discussion is conducted quarterly. During these meetings, the linkage manager reinforces recovery if the patient is not using drugs or alcohol or facilitates treatment re-linkage if necessary.

MI techniques are a key part of the intervention, and they are used during checkups to move individuals along the stages of change (e.g., pre-contemplation, contemplation, preparation, action, maintenance) believed necessary to prepare them to take action related to substance misuse (17). Using MI, linkage managers provide feedback, cultivate change talk, develop a sense of partnership between linkage managers and patients, and express empathy. Linkage managers must complete extensive MI training and achieve and maintain a pre-determined level of MI competency using professionally recognized standards (18).

As described above, RMC-PC is a complex intervention, and complex interventions developed for and found effective through research often encounter difficulties translating to real-world practice, thus limiting their public health potential (19–22). RMC-PC, and all other iterations of RMC, have only been delivered by research staff working under highly controlled conditions. Therefore, testing RMC-PC’s implementation and effectiveness in primary care practice is the next logical step in the intervention’s maturation. We conducted a feasibility study to inform the selection of an implementation strategy for a future full-scale RMC-PC hybrid effectiveness-implementation trial. Our aims were to identify (1) implementation determinants (barriers and facilitators) to RMC-PC within an FQHC setting and (2) differences between an FQHC linkage manager (F-LM) and a research staff linkage manager’s (R-LM) RMC-PC delivery. As aligned with the recommended area of inquiry for feasibility studies (23–25), questions guiding this work included: To what extent does RMC-PC fit an FQHC’s A/SUD service needs?; To what degree can a primary care staff person deliver RMC-PC as intended?; What determinants of implementation can support or hinder RMC-PC implementation in a high-volume primary care setting? We decided to focus on these questions because our prior clinical trials have already provided information beyond what could be gained in a pilot or feasibility study for informing the feasibility of recruitment and retention procedures, specific data collection instruments and protocols, and sample size calculations (12, 13, 23).

## 2 Methods

We used a convergent mixed method design in which quantitative and qualitative data are collected and analyzed separately, and then results are combined to develop overall conclusions (26). We used this approach to obtain qualitative implementation determinant data to complement and expand the knowledge gained from quantitative results. Mixed method designs are recognized for optimizing learning from feasibility studies by developing meta-inferences about implications for future, larger-scale work (24, 25, 27). All research procedures described were approved by Chestnut Health System’s Institutional Review Board (#1160–0122).

### 2.1 Study setting

The study’s setting was a Federally Qualified Health Center (FQHC) located in a large midwestern city without any prior RMC-PC experience. The site has an internal behavioral health program, including robust medication-based treatment for patients with alcohol and opioid use disorders. The FQHC also had prior experience conducting SBIRT for mental health disorders, but none conducting it for A/SUD risk. However, the FQHC had a goal of implementing A/SUD SBIRT screening, we offered to assist with this to establish the screening process that would feed into RMC-PC for the feasibility study.

### 2.2 Intervention and modifications

One of two linkage managers administered the intervention: (1) the F-LM was a behavioral health care coordinator with a master’s degree in social work, and (2) the R-LM had an associate degree and 3 years of experience delivering RMC services. The R-LM also worked off-site from the FQHC and was assigned to two other RMC projects. Both linkage managers delivered the following key RMC-PC components: (1) baseline patient assessment; (2) baseline linkage meeting; (3) case tracking activities (to ensure patients could be located for the final linkage meeting); (4) regular patient check-ins; and (5) a final 30-day linkage assessment. RMC has been delivered effectively using both in-person and virtual modalities. While the F-LM met all patients at least once in person during recruitment, patients could complete baseline and follow-up assessments in person or by phone. The R-LM only interacted with patients by phone.

This approach included three modifications from the standard RMC-PC intervention to accommodate the FQHC context. First, in prior clinical trials, assessments and case tracking were completed by research assistants, which is not possible in real-world practice. Second, all activities were completed within 30 days vs. 12 months to reduce the FQHC’s burden related to redirecting a staff person to perform LM activities. Finally, due to challenges the FQHC experienced implementing SBIRT screening (see below), linkage managers worked with patients already scheduled to begin treatment vs. those not engaged. These last two modifications were reasonable given (a) a 30-day version of RMC has been delivered to patients already starting A/SUD treatment with promising retention results (28) and (b) this approach required the linkage managers to carry out those tasks with the most potential practice translation barriers.

### 2.3 Training approach

Both the F-LM and R-LM completed two core training activities. First were 38-hour training sessions, including one session on MI principles and two on applying these principles using the linkage worksheet. The training component included supplemental readings, didactic and interactive components, opportunities for reflection, and practice activities. Second, they listened to recorded linkage meetings and practiced applying MI with coached feedback. The instructor and coach was a nationally certified MI trainer who had trained linkage managers in prior RMC research (29). These activities were delivered using a virtual format. However, both linkage managers had prior education and experience applying MI to facilitate their training. As the delivery of the intervention by FQHC staff requires linkage managers to take on duties previously carried out by research assistants, both linkage managers also completed a one-hour training on the collection of data to inform the linkage worksheet and watched a one-hour recorded case tracking video explaining the process for locating and staying in touch with patients.

### 2.4 Patient recruitment

Based on the initial timeline of activities, we planned for SBIRT screening to be fully running for at least 1 month before the start of linkage services and feed into RMC-PC recruitment. However, the FQHC could not implement SBIRT within the originally projected 3-month window due to difficulties integrating separate AUD (30) and SUD (31) screening instruments within their new electronic health record system—an issue more fully discussed in the results section. While we gave them an additional 3 months, we were required to move ahead with the study before the linkage managers would require retraining in intervention activities. Therefore, we expedited an alternative plan to assess linkage managers’ ability to conduct linkage meetings and check-ins among patients arriving for their first A/SUD treatment appointment. This modified approach was still considered valuable because the aim was to understand F-LM’s ability to complete the activities specific to the linkage meeting process and because we already had a strong understanding of how the screening to RMC handoff worked from the previous trials (8–12).

### 2.5 Data and procedures

#### 2.5.1 Screening

Prior to beginning any implementation activities, we conducted A/SUD risk screening to establish an understanding of the potential rate of referral from primary care visits to RMC-PC. Two medical assistants conducted the screenings with all patients seen as part of rooming procedures over 5 business days. We assessed risk using 6 questions from the Substance Use Problem Scale of the Global Assessment of Needs Short Screener (GAIN-SS) (32). These questions asked, “When was the last time…”

1. You used alcohol or other drugs weekly or more often?
2. You spent a lot of time either getting alcohol or other drugs, using alcohol or other drugs, or recovering from the effects of alcohol or other drugs?
3. You kept using alcohol or other drugs even though it was causing social problems, leading to fights, or getting you into trouble with other people?
4. Your use of alcohol or other drugs cause you to give up or reduce your involvement in activities at work, school, home or social events?
5. You had withdrawal problems from alcohol or other drugs like shaky hands, throwing up, having trouble sitting still or sleeping, or you used any alcohol or other drugs to stop being sick or avoid withdrawal problems?
6. You received treatment, counselling, medication, case management, or aftercare for your use of alcohol or any other drug? Please do not include any emergency room visits, detoxification, self-help or recovery programs.

These questions provide a count of past year symptoms related to any A/SUD. The instrument has the following established cut points for A/SUD risk: 0/“low,” 1–2/“moderate,” 3–6/“high.” A score of 1 or higher would indicate potential eligibility for RMC-PC services. Medical assistants administered all instruments electronically using a web-based portal connected to a tablet computer. This screening process was discontinued after five business days because it was too difficult for them to manage using the external system and because SBIRT screening forms were scheduled to be integrated within the electronic health record workflow before starting RMC-PC patient recruitment. As described above, SBIRT was not implemented so we alternatively enrolled patients at their first treatment appointment. Because these patients already had a diagnosed SUD, no screening was required to assess RMC-PC eligibility.

#### 2.5.2 RMC linkage data

We used several data points to compare linkage managers’ performance, which are standard metrics collected in RMC studies (8–13). These include the rate of successful linkage meeting (baseline and follow-up) completion; days between patient consent to participate in the study and baseline linkage meeting; days between baseline and follow-up linkage meetings; the number of days on which linkage managers attempted to contact patients between baseline and follow-up linkage meetings; and an assessment of recorded meetings of linkage manager motivational interviewing performance. Patients arriving for an initial A/SUD treatment appointment were informed of the study and consented by the FQHC linkage manager.

Assignment of patients between the F-LM and R-LM alternated, with recruitment occurring until both linkage managers completed five recorded baseline linkage meetings. The F-LM alerted the research team immediately after receiving a patient’s consent and completed or scheduled the initial linkage meeting depending on patient availability. For patients assigned to the R-LM, FQHC staff sent their contact information directly to the R-LM who immediately attempted to contact them to complete the linkage meeting by phone. At the beginning of each meeting, linkage managers completed a shortened version of the GAIN-SS that included the Substance Use Problem Scale. They then moved directly from the assessment to the linkage meeting. All attempts to contact patients after consent were recorded in an Excel spreadsheet. To prevent overburdening the F-LM who was adding these activities to her regular job duties, we planned to stop enrollments for each linkage manager after they had completed five successful baseline meetings. All linkage meetings were recorded.

#### 2.5.3 Implementation determinants

The lead author conducted a 90-min focus group with the FQHC’s Director of Behavioral Health, the Quality Nurse Manager who oversaw the planned, but ultimately not completed, SBIRT implementation, one SBIRT screener, and the F-LM. We developed questions using the Consolidated Framework for Implementation Research (CFIR) as a guide (33). CFIR comprises 37 constructs reflective of implementation determinants across five domains: (1) the intervention’s defining characteristics, (2) the inner setting (i.e., the environment in which the intervention is being implemented), (3) the outer setting (i.e., the environment existing outside of the implementing organization), (4) characteristics of individuals involved in the implementation, and (5) the process that facilitated the implementation. Some example questions from the interview include: “How complicated do you feel RMC’s components are to deliver?”; “Please tell me what specific infrastructure, technology, staffing or resources could have improved your pilot implementation of SBIRT and RMC?”; “Can you discuss any state or federal policies or laws that would impact the delivery of the RMC intervention?”; “Can you tell me about your experiences implementing SBIRT and RMC after trainings were completed?” The interview was recorded and transcribed.

### 2.6 Analysis

#### 2.6.1 Quantitative

Following recommended guidelines for feasibility study reporting, only descriptive statistics are presented (34, 35). We calculated frequencies for characteristics of patients who completed GAIN screening. To compare F-LM and R-LM’s performance, we calculated percentages of patients assigned, linkage meetings completed and days from enrollment to baseline linkage completion, days on which successful checkups were completed, and days from baseline to follow-up linkage meeting completion. We calculated effect sizes for completion rates (odds ratios) and days between checkups and successful linkage completion (Cohen’s d).

#### 2.6.2 Qualitative

The MI coach reviewed all linkage meeting recordings. The MI coach (a member of the Motivational Interviewing Network of Trainers) assessed the quality of the discussions using MI delivery guidelines she developed as part of her training and consulting practice and had since adapted for use with RMC. These guidelines were used based on her expertise and familiarity with other MI fidelity tools, which she indicated were inappropriate for assessing a linkage meeting that combined the closed-ended GAIN-based assessment with MI techniques. The coach’s guidelines include (a) whether the linkage manager consistently and correctly used the two “rulers” that ask patients to rate (1) their perceived importance of receiving treatment and (2) their confidence in engaging in treatment on a scale of 1–10; (b) use of strategies to engage patients in the discussion and evoke change talk; and (c) overall demonstration of the linkage manager’s commitment to the spirit of MI through demonstration of compassion, acceptance, partnership, and empowerment.

The lead author conducted a hybrid deductive-inductive analysis of the focus group transcript (36). This approach was chosen for its pragmatic utility in addressing the specific research questions (37). Coding was performed using the comments function in Microsoft Word. The Consolidated Framework for Implementation Research (CFIR) was used as the *a priori* coding framework for the deductive component (33). Given that CFIR constructs are general in nature, subcodes were developed inductively to better capture the specific determinants within the implementation context. The coded segments were then labeled as either a facilitator or a barrier. Segments were then reviewed by codes to identify any potential patterns and particularly salient points made by focus group participants. This approach enabled a nuanced analysis that remained grounded in the theoretical framework while being responsive to the particularities of the data. Due to the limited scope of the data, thematic saturation was considered to have been achieved when no new insights emerged upon further review of the transcript (38).

The lead author developed a matrix to compare quantitative results and qualitative findings to develop meta-inferences and conclusions (26). This matrix was shared with the fourth author (one of the RMC model developers) and the fifth author (an experienced RMC-PC researcher). They discussed the data mixing results and their potential implications for a hybrid RMC-PC study.

## 3 Results

### 3.1 A/SUD screening rates

Table 1 displays demographics and A/SUD risk scores for 58 patients screened over **5** days. No patients were reported to have refused screening. Demographic characteristics were similar to those of the FQHC’s larger population in that there were high percentages of patients between 25 and 65 years of age (range = 18–64; mean = 44.19; sd = 13.4) and were majority female, Black, and non-Hispanic. Half of the patients screened had a score indicating moderate to high risk for an A/SUD diagnosis, indicating eligibility for RMC linkage services if they were available then.

**Table 1:** Patient demographics and alcohol/substance use disorder (A/SUD) risk scores.

	Frequency	Percent
Age		
18–24	5	9
25–34	10	17
35–44	14	24
45–54	13	22
55–64	16	28
Gender		
Female	30	52
Male	26	45
Other	2	3
Race/Ethnicity		
Black	39	67
White	6	10
Other	13	23
Ethnicity		
Hispanic/Latino	9	16
Non-Hispanic/Latino	49	84
A/SUD risk		
Low	29	50
Moderate	22	38
High	7	12
Total	58	100

### 3.2 Comparison of linkage manager performance

Table 2 compares the demographics of patients who: (a) were assigned to linkage managers after indicating initial interest in RMC-PC services and (b) completed an initial linkage meeting. There were no considerable demographic differences between patients initially assigned to each linkage manager. However, the R-LM was assigned two additional patients because she had less success locating patients and completing the quota of five baseline linkage meetings after the initial patient assignment was made. The F-LM was assigned relatively older and more female and White patients. They had a higher rate of baseline linkage meeting completion overall and with all demographic categories except males, among whom they had a slightly lower rate of baseline meeting completion. Finally, the F-LM had a greater proportion of clients with any substance use in the 30 days before the baseline linkage meeting.

**Table 2:** Comparison of demographic characteristics of patients by initial linkage manager assignment and baseline linkage meeting completion.

		Assigned			Baseline linkage meeting completed
		F-LM (*n* = 7)	R-LM (*n* = 9)	F-LM (*n* = 7)^a^	R-LM (*n* = 9)
All		7 (100%)	9 (100%)	6 (86%)	5 (56%)
Age					
	35–44	2 (29%)	3 (33%)	2 (100%)	2 (67%)
	45–54	1 (14%)	3 (33%)	1 (100%)	1 (33%)
	55+	4 (57%)	3 (33%)	3 (75%)	2 (67%)
Gender					
	Female	4 (57%)	2 (22%)	4 (100%)	0 (0%)
	Male	3 (43%)	7 (78%)	2 (67%)	5 (71%)
Race					
	Black	5 (71%)	7 (78%)	4 (80%)	5 (71%)
	White	2 (28%)	2 (22%)	2 (100%)	0 (0%)
Any use in past 30 days				F-LM (*n* = 6)^a,b^	R-LM (*n* = 5)^b^
	Any substances	–	–	6 (100%)	3 (60%)
	Alcohol	–	–	2 (33%)	3 (60%)
	Marijuana	–	–	0 (0%)	1 (20%)
	Cocaine	–	–	3 (50%)	2 (40%)
	Other stimulants	–	–	1 (17%)	0 (0%)
	Opioids	–	–	6 (100%)	2 (40%)

All patients were non-Hispanic/Latino; ^a^an extra patient was enrolled because of the interview recording failed; ^b^substance use only assessed if patient completed baseline linkage meeting.

Table 3 compares linkage managers’ rates of baseline and follow-up linkage meeting completion. The F-LM had almost five times the odds of successful baseline linkage meeting completion and twice the odds of 30-day follow-up meeting completion. Both linkage managers had one patient who declined the linkage meeting after initially indicating interest in RMC services and the R-LM had three patients who could not be reached to complete baseline linkage. All incomplete follow-up linkage meetings resulted from linkage managers’ inability to reach the patient.

**Table 3:** Comparison of linkage manager baseline and follow-up linkage meeting completion rates.

	F-LM			R-LM			
	Assigned	Completed	Rate	Assigned	Completed	Rate	OR
Baseline	7	^a^6	86%	9	5	56%	4.80
Follow-up	7	5	71%	9	5	56%	2.00

^aAn extra patient was enrolled because of the interview recording failed.^

Table 4 shows that the effect of linkage manager assignment related to the speed of baseline linkage completion was large, with average days until baseline meeting completion (1.33 days F-LM vs. 4.4 days R-LM; *d* = −1.08) and a small effect was observed for the speed of follow-up meeting completion (56 days F-LM vs. 59 days R-LM; *d* = −0.20). No effect was observed for the number of successful check-ins completed.

**Table 4:** Comparison of days elapsed between engagement and baseline and baseline and follow-up meetings and number of successful check-ins between baseline and follow-up meetings.

		F-LM		R-LM		Overall		
		Mean	SD	Mean	SD	Mean	SD	Cohen’s d
Days from enrollment to baseline linkage meeting		(*n* = 6)^a^		(*n* = 5)		(*n* = 11)		
		1.33	1.63	4.4	3.21	2.73	2.83	−1.08
Number of days on which successful check-ins were completed		(*n* = 6)		(*n* = 5)		(*n* = 11)		
		2.67	1.21	4	3.16	11.02	3.27	−0.12
Days from baseline to follow-up linkage meeting		(*n* = 5)^b^		(*n* = 5)		(*n* = 10)		
		56	10.61	59	20.14	57.5	15.26	−0.20

^aAn extra patient was enrolled because of the interview recording failed.^

^bOnly data from patients who completed a baseline were used to calculate follow-up days.^

### 3.3 Linkage meeting discussion quality

The MI coach’s assessment of the linkage meeting discussions demonstrated that both linkage managers missed opportunities to use evoking strategies and change talk during their linkage meetings. This was because linkage managers would often not probe related to questions for which they had already received a closed-ended answer during the assessment. However, the R-LM consistently offered follow-up questions related to the importance and confidence of rulers, whereas the F-LM did not. Yet, the F-LM more consistently displayed MI spirit (i.e., the relational aspects of MI that convey compassion, acceptance, partnership, and empowerment) through tone of voice and transparency about notes they took during meetings.

### 3.4 Implementation determinants identified

The focus group data identified 11 determinants divided among 4 domains that were believed to have impacted RMC-PC implementation in the FQHC setting. The determinants are listed in Table 5 by domain with example quotes. As an innovation (Domain 1) within the setting, RMC-PC was considered to have a relative advantage over care as usual because it provided more patient support and resources and because the more intensive and consistent contact was believed to have the potential for improving patients’ retention in A/SUD services (particularly those for opioid use disorder).

**Table 5:** RMC-PC CFIR-mapped implementation determinants identified by focus group participants.

CFIR domain	CFIR construct	Barrier or facilitator	Specific determinant	Example quote
Innovation	Relative advantage	Facilitator	Intervention offers more support and resources than care as usual	I [the linkage manager] was making more of an impact with them [using an RMC model vs. care as usual] as well…the resources and the help that I was able to provide for them to be able to constantly make the appointments or not have that as a worry, or barrier, to coming into the appointments.
Innovation	Relative advantage	Facilitator	More intense and consistent patient contact can lead to better treatment retention	With others [interventions], it’s not as often. So, it’d be with the RMC here, or in general, it’s your contact or follow up with the participants should happen more often versus other times […] Respondent: And so, I think from other interventions that we have done, … having that same person [reaching out to contact them] is helpful.
Inner setting	Mission alignment	Facilitator	RMC-PC is compatible with mission	Interviewer: To what extent do you think that RMC is compatible with [the FQHC’s] mission, goals, and values? Respondent 1: I think it’s on point. Respondent 2: [We] want to provide concierge service to the patients who need it the most has been my goal and it gets us closer to that.
Inner setting	Available resources	Barrier	Technology department did not have resources to implement screening within ERH in timely manner.	Respondent: We have the full build, so, everything is embedded in [the electronic health record]… Interviewer: How many months did that take to get built? Respondent: I think a year. Interviewer: Originally [they were supposed to have it done in] 3 months? Respondent: Yeah, they were very apologetic.
Inner setting	Compatibility	Barrier	The SBIRT screening process was incompatible with the clinical workflow	Everyone has to be involved [in the SBIRT process], from front desk to operations… I think the other hard part is, if we do not embed the actual process with our workflows, it’s not going to be sustainable. And I think that that was, like, the even harder part….
Individuals	Innovation recipients (need)	Facilitator	Patients prefer a single point of contact	Knowing that they [patients] have the one contact person is a positive thing for them to keep the engagement going, because, like you said, when they, that relationship is broken or something changes about it, it affects them in their recovery and wanting to keep coming. They do not want to have to get to know another person or build rapport with another person and they lose focus. So, that is a big, that’s a big one for our population here.
Individuals	Innovation recipients (need)	Facilitator	Patients want linkage to services	And they [patients] actually, they, kind of, asked me [linkage manager], like, “Is there anything else [additional services] for substance use going on that I could take advantage of?”
Individuals	Innovation deliverers (motivation)	Facilitator	Feeling of helpfulness motivated the linkage manager	I like to feel helpful. And to feel like I’ve accomplished something. And to also feel like I’ve been a part of somebody else’s change.
Individuals	Innovation deliverer (opportunity)	Facilitator	The linkage manager able to do RMC-PC work alongside regular duties	I feel like I was able to still maintain my regular workflow.
Individuals	Other implementation support (motivation)	Barrier	Staff burnout	…being understaffed, people being burnt out because of COVID, and tired is another thing. Both, all staff from front desk, providers…Quality fatigue, unfortunately, is [another] issue because we have so many quality metrics, we are trying to improve without ever increasing the visit time for patients. So, doing more with the same amount of patient volume, with less support staff.
Outer setting	Financing	Barrier	RMC-PC services are not covered under traditional FQHC reimbursement mechanisms	Most FQHCs, we do not, we just bill through the medical system. So, there’s no way to, that’s the other thing, is allowing medical to reimburse for substance use interventions, especially something like this, because it’s supposed to be done in the primary care setting, right? So, why cannot we get reimbursement on this side of it?

Regarding the inner setting (Domain 2), while RMC-PC was stated to be highly aligned with the FQHC’s mission, implementing SBIRT as a screening process for linkage referral was a barrier. Specifically, the SBIRT process could not be implemented within the study period for two reasons. First, the Information Technology Department did not have the time or resources to integrate the SBIRT screeners into the electronic medical record within the three-month time span they originally quoted. The second related reason was that, without electronic health record integration, the SBIRT process was incompatible with the examination workflow. Specifically, paper-based screening could not be completed within the short fifteen-minute window in which a patient is in the examination room.

Several determinants related to individuals were identified (Domain 3). Patients were perceived to prefer having a single point of contact that can simplify their interactions with providers, which would ultimately improve treatment engagement. The F-LM also commented that patients she had served asked to be connected to additional recovery support services, indicating a need and desire for A/SUD treatment linkage and supports. The F-LM also indicated that delivering RMC-PC services made her feel as if she could provide a higher level of assistance to patients than she previously had and that this provided her with a sense of motivation. She also believed that RMC-PC-related activities were compatible with her individual workflow and did not interrupt her ability to accomplish her regular tasks. Despite this, it was recognized that burnout was a barrier related to other staff involved in the implementation because they had been dealing with high levels of stress during the COVID-19 pandemic that was starting to ease and because they are always having items added to their workflows due to the many quality metrics FQHCs are required to report.

Finally, the largest barrier to implementation identified was related to the outer setting (Domain 4). Implementation team members did not have a way to bill for RMC-PC services. While FQHCs are consistently encouraged to deliver integrated primary and behavioral health care, there are currently no mechanisms in place to support such services adequately. As such, it was perceived that RMC-PC could not be implemented or sustained without grant funding.

## 4 Discussion

Table 6 presents a crosswalk of quantitative results and qualitative findings as they pertain to each research question, as well as the final conclusions drawn by comparing them. We expand on these conclusions and their relevance to future RMC-PC research below.

**Table 6:** Crosswalk of primary quantitative and qualitative results/findings and conclusions for each research question.

	Quantitative results	Qualitative findings	Meta-inferences/conclusions
Question			
To what extent does RMC-PC fit an FQHC’s A/SUD service need?	More than half of patients screened with the GAIN-SS were identified as having moderate to high A/SUD risk Majority of patients completed baseline and follow-up linkage meetings	Highly compatible with FQHC mission. Patients have a preference for single-point contact More intensive contact addresses A/SUD service retention needs F-LM relayed patient requests for additional A/SUD services	RMC-PC is compatible with the FQHC’s mission and patient service needs and preferences.
To what degree can a primary care staff person deliver RMC-PC as intended?	F-LM had greater: ○ Proportion of patients with recent substance use ○ Observed effect for baseline and follow-up linkage meeting completion ○ Observed effect for days to baseline and follow-up linkage meeting	Compared to R-LM, the F-LM displayed: ○ Stronger MI spirit ○ Less follow-up probing ○ Similar difficulties evoking change talk ○ F-LM was able to perform RMC-PC activities alongside regular duties	The F-LM managed the RMC-PC workflow in addition to normal duties while performing at a level comparable to or slightly higher than the R-LM in linkage meeting completion and MI performance despite having clients with more recent substance use.
What determinants of implementation can support or hinder RMC-PC implementation in a high-volume primary care setting?	F-LM completion was higher than R-LM.	Facilitators included RMC’s compatibility with FQHC mission, needs, patient and F-LM preferences, and F-LM workflow Barriers included: ○ Closed-ended linkage meeting questions impact on MI performance & lack of fit with MI and assessment questions ○ SBIRT screening was not able to be implemented on proposed timeline because: lack of technological resources, incompatibilities with primary care workflow, staff burnout ○ Lack of billing mechanisms to support RMC-PC	RMC-PC was largely compatible with the setting, and the LM was able to carry out the work. However, barriers at the organizational and individual level delayed SBIRT implementation, so an alternative plan had to be pursued. Additionally, integration assessment protocols into the linkage meeting negatively impacted MI. The ability to financially support RMC-PC is a barrier that will be encountered outside of research.

RMC-PC was largely compatible with the FQHC’s mission and service needs. FQHCs, in general, have a mission to serve the most medically, economically, and socially vulnerable populations, placing them at the forefront when it comes to identifying and implementing innovative integrated care models (39, 40). About half of patients who completed GAIN-based screening during a primary care visit had substance use risk scores indicating a need for RMC-PC services. This rate is higher than the 24% positive A/SUD screening rate observed in our prior RMC-PC clinical trial (13). Among the 16 patients who were offered RMC-PC services, the majority completed baseline and follow-up meetings (69 and 63%, respectively), indicating the intervention was likely acceptable to them. This was reinforced in the focus group with management and staff, which indicated the linkage manager role addressed patients’ preference for a single point of contact when navigating care. The preference for a single point of contact and its benefits is reinforced by the literature on integrated primary care and the management of chronic illnesses (40–45), which includes A/SUD (46). The focus group also indicated that RMC-PC could help address low retention rates for opioid use disorder treatment, which hover around 43% nationally (47).

Results indicate it is potentially feasible to expect an F-LM to deliver RMC-PC at a level comparable to a R-LM if they are provided proper support. Indeed, the F-LM performed higher in some areas than the R-LM. For instance, the F-LM had greater success completing baseline and follow-up linkage meetings. As the R-LM was identified as working for an external organization, this finding might have to do with patients’ preference for receiving integrated A/SUD services from a primary care site, which is supported by prior research (48, 49). It is also possible that the F-LM’s ability to meet patients in person helped them build stronger relationships that improved interactions and follow-up than the R-LM was able to over the phone. Meeting patients in person might have also made it easier for the F-LM to invoke MI spirit, and this improved MI spirit might have accounted for her higher linkage manager completion rate given her RMC-PC caseload was composed of patients with more recent substance use, indicating a potentially lower commitment to change (50). The F-LM also accomplished this while continuing to perform her regular job duties, suggesting RMC-PC activities can be performed alongside FQHC staff person’s other responsibilities if needed. It is essential to recognize that the R-LM, while more seasoned and having a high level of prior MI performance, was working under limiting conditions compared to her usual work, having taken on activities generally performed by a research assistant and delivering services solely by phone. This has implications when considering the benefits and drawbacks of potential embedded vs. external approaches to implementing RMC-PC in practice.

The primary implementation barriers identified to be cognizant of in future RMC-PC work include the screening process used to identify RMC-PC-eligible patients, the incompatibility of assessment procedures with MI, and the lack of adequate financial support for RMC-PC outside of the research context. Strong clinic workflow and electronic health record integration are noted facilitators of the SBIRT implementation (44, 46, 47), and most of the barriers to integration we encountered focused on the SBIRT screening process. Since the RMC model does not require SBIRT, it is possible that these issues could have been reduced if the FQHC had continued to use the GAIN A/SUD screener beyond the initial two-week screening activity. Comprising only five questions to assess A/SUD risk, the GAIN is much shorter than the combined AUDIT (for AUD) and DAST (for SUD) screeners, which can take up to 22 questions to complete when combined with an initial two-question screener (51). As such, the GAIN could considerably reduce the length of screening associated with instruments SBIRT commonly utilizes, which are generally estimated to add up to 5 min to an already tight clinic workflow (52). We do not expect this to be an issue, as SBIRT is considered to be adaptable to the needs of a local setting (53, 54). The GAIN may be more sensitive, potentially leading to a higher rate of false positives, as indicated by positive screening rates that were twice as high as those found in the previous RMC-PC clinical trial using the AUDIT and DAST (13). Therefore, cross-validation of the GAIN with these more widely used screening instruments is suggested before it is used in primary care screening. SBIRT implementation issues we encountered would not exist in an FQHC with an already functioning SBIRT program, and the focus of implementation in such sites would be replacing an existing SBIRT referral component with RMC-PC. In clinics without a pre-established SBIRT workflow (or one that is not functioning optimally), RMC-PC could be implemented using an alternative screening and referral model.

Both linkage managers had difficulties facilitating open-ended MI discussions after completing the closed-ended assessment questions in the linkage worksheet. This is because assessment questions likely reinforced MI-inconsistent behaviors (e.g., monotone voice, verbatim question reading, failure to probe or evoke change talk) that affected discussion quality (55, 56), and similar findings have been identified in survey research when interviewers do not sufficiently administer open-ended questions when they feel closed-ended responses have already answered them (57). A possible solution is that linkage managers could be trained to use an MI sandwich approach in which a standardized assessment is sandwiched between an MI-focused discussion of problems and an MI discussion of change (58). However, this second approach would require modifying RMC-PC’s current MI training curriculum and fidelity assessment standards. A second approach that could be easily accomplished is if A/SUD screening questions are administered electronically by programming the instrument to ask assessment questions if the patient receives a moderate to high A/SUD risk score, prior to the linkage meeting.

Funding poses an issue for both the implementation and sustainability of RMC-PC. Medicaid reimbursement policies that prevent reimbursement for physical and behavioral health services delivered on the same day have been identified as a primary barrier to care integration (59). For RMC-PC, this prevents reimbursement linkage services from occurring on the same day as SBIRT screening, which is necessary for the model’s preferred immediate warm handoff approach. Rules that restrict billable encounters to licensed staff limit the use of capable and less expensive paraprofessional staff as linkage managers (60–63). While some FQHCs have developed successful approaches for supporting care coordination through strategic implementation of value-based performance incentives, such approaches can be limited by state-level Medicaid policies (63, 64). Given current service funding limitations, we plan to include a payor and provider advisory board in future RMC-PC studies. The goal of this board will be to assist in identifying innovative funding approaches and data necessary to change federal and state policies limiting funding of RMC-PC and other effective integrated care approaches.

As a next step in this work, we are developing a packaged implementation strategy to improve RMC-PC’s implementation and sustainment feasibility. The SBIRT literature points to several strategies that can be used to improve the implementation of A/SUD screening, which we are investigating. Some of the most endorsed include embedding reminders in the electronic health record, task shifting, and summary reports/dashboards (54). However, one of the most useful strategies to use in screening naïve settings, given the current study’s results, is likely to conduct a baseline needs assessment that would include electronic health record integration readiness (54). Effective RMC linkage relies on LMs having high MI proficiency, and training and coaching supports, as have been delivered in our prior research endeavors, are time-consuming, expensive, and difficult to scale. In response, we are developing a more efficient package of training and coaching tools that include asynchronous eLearning modules and an artificial intelligence coaching program that can provide real-time feedback (65–67). As previously stated, we are also developing a payor and provider advisory board to help identify financial strategies to support RMC-PC’s implementation and sustainment. It is hopeful that national efforts to support integrated/coordinated care activities and peer support specialists and health workers (who are in ideal positions to serve as LMs) through Medicaid and Medicare billing present potential solutions to recognized financing issues (63, 68).

### 4.1 Limitations

This study was conducted at a single site within a limited timeframe, and, likely, we did not identify all potential issues associated RMC-PC implementation and delivery that will be encountered in future work. The small sample size (regarding both site and patients) also prevents us from making any conclusions regarding implementation or client outcomes. While some feasibility work is focused on identifying effect sizes to inform future power calculations, this was unnecessary given the recently completed RMC-PC clinical trial already provides this information (13). Because of the barriers to implementing SBIRT, the RMC recruitment pathway was not optimal. Therefore, we are unsure if results would have been similar if SBIRT had been in place. With only two linkage managers, it is impossible to know if differences in performance were connected to their positions as FQHC or research staff or if variations were related more to personal attributes. We are also unsure whether the difference in LM modality (in-person vs. phone) confounded outcomes. Despite these limitations, the study led to several new insights beyond those obtained in our prior clinical trial (13) that will be critical in moving RMC-PC into real-world practice, thereby accomplishing our primary goal of this feasibility study.

## 5 Conclusion

RMC-PC offers a potential strategy for addressing recognized deficiencies in A/SUD treatment referral from primary care settings; however, the intervention has yet to be translated into practice outside of rigorously controlled clinical trials. This study provides preliminary evidence that RMC-PC is feasible for FQHC staff to deliver while identifying several factors that must be considered to ensure the successful implementation and sustainment of the intervention. Using this knowledge will be critical to finalizing a packaged implementation strategy that we plan to use and test in a future hybrid effectiveness-implementation trial. The current study also demonstrates that funding is one of the most important barriers to the uptake, implementation, and long-term sustainability of RMC-PC within an FQHC and that future research is important for identifying potential financial support mechanisms.

## Data Availability

The de-identified data supporting the conclusions of this article will be made available by the authors, without undue reservation.
